# Prevalence and Risk Factors of Asymptomatic Gallstone Disease in North-East Part of Bangladesh

**DOI:** 10.5005/jp-journals-10018-1118

**Published:** 2015-01-06

**Authors:** Madhusudan Saha, Kamrun Nahar, MM Arif Hosen, MH Khan, Shasanka Kumar Saha, Bimal Chandra Shil, Md Habibur Rahman

**Affiliations:** 1 Department of Gastroenterology, North East Medical College, Sylhet, Bangladesh; 2 Nuclear Medicine and Ultrasound Center, Sylhet, Bangladesh; 3 Department of Gastroenterology, Sir Salimullah Medical College, Dhaka, Bangladesh

**Keywords:** Asymptomatic gallstone, Prevalence of gallstone disease, North-East Bangladesh.

## Abstract

**Background:**

The study was designed to assess the prevalence of asymptomatic gallstone disease in North-East part of Bangladesh.

**Materials and methods:**

Randomly selected asymptomatic persons, unknown to have gallstone disease, from both rural and urban areas were enrolled. They were subjected to abdominal ultrasonography and findings were recorded in a data sheet.

**Results:**

Total 1,019 persons (316 males and 703 females) were examined. Age of them varied from 18 to 80 years with mean age of 37.22 years. Out of them, 61 (6%) persons were found to have gallstone. Among them, 14 were males and 47 were females. Both male and females of age below 40 years were more affected. Gallstone disease was found more commonly among housewives and middle class people. Among 61 patients with gallstone, seven were underweight (11.47%), 32 (52.45%) had normal weight and 22 (36.06%) were overweight, obese or extremely obese. But, this difference was not statistically significant (p = 0.894).

**Conclusion:**

Prevalence of asymptomatic gallstone disease was found in 6% apparently healthy subjects of North-East part of Bangladesh. It was more prevalent among housewives and middle class group of population. It is also common among the people of age group below 40 years.

**How to cite this article:**

Saha M, Nahar K, Hosen MMA, Khan MH, Saha SK, Shil BC, Rahman MH. Prevalence and Risk Factors of Asymptomatic Gallstone Disease in North-East Part of Bangladesh. Euroasian J Hepato-Gastroenterol 2015;5(1):1-3.

## INTRODUCTION

The prevalence of gallstone disease (GSD) shows geographical variations. It occurs commonly in western countries. ^1,2^ Most are asymptomatic, but still, GSD contributes substantially to healthcare cost and its complications are sometimes life-threatening.^1-3^ Epidemiological studies show that 80% subjects with GSD are asymptomatic.^1-4^ The prevalence of GSD is 15% in Sweden,^5^ 14.5% in Mexico;^6^ while, in Italy, it is 5.9%.^7^ In Asian countries, Taiwan has the highest prevalence of GSD at 20%.^8^ Whereas the prevalence of GSD in Japan and Thailand are 3.2 and 3.5% respectively.^9,10^ In India, the prevalence rate is 0.00 to 8.1% in men and 2 to 29% in women.^11^ Asymptomatic GSD is being increasingly detected nowadays as result of the widespread use of ultrasonogram.^12^ Real-time ultrasonogram is a simple, noninvasive and relatively less costly method for diagnosis of GSD with sensitivity up to 95.5%.^13^ In Southern coastal region of Bangladesh, one study was done among rural people showing overall prevalence of gallstone disease at 5.4% (7.7% in women and 3.4% in men).^14^ This study was done to see the prevalence of asymptomatic GSD in the North-East part of Bangladesh.

## MATERIALS AND METHODS

This study was done between January 2012 and December 2012 in the Centre of Nuclear Medicine and Ultrasound, Sylhet, Bangladesh. Randomly selected apparently healthy people of age 18 years and above from Sylhet city and adjacent villages were included in this study. Any person known to have gallstone disease or previously investigated for gallstone disease was excluded. Epide-miological data and physical findings were recorded in a data sheet. Then, sonological examination was done by two senior sonologists of the center separately using Machine Sonoline G60 S (Company Siemens, Erlangen, Nuremberg, Germany). In case of doubt, the person was examined by senior most sonologist of the center. Around five persons were examined at each working day. Criteria for diagnosis of GSD were as follows:

- One or more echogenic structures in gall bladder lumen with posterior acoustic shadow.
- One or more echogenic structures without posterior acoustic shadow but changing position during changes of posture.
- A strongly echogenic structure in the region of gallbladder with distal acoustic shadow in case of limited or nonvisualization of lumen.

## STATISTICAL ANALYSIS

Statistical analysis was done by using SPSS version 12 and χ^2^ test was done to see significance. p-value < 0.05 was taken as significant.

## RESULTS

Total 1,019 persons were enrolled in this study. Among them, 316 (31%) were males and 703 (69%) were females. Age of them varied from 18 to 80 years (mean 37.22 years). Out of them, 402 participants (123 males and 279 females) were from rural areas and 617 (195 males and 424 females) were from urban area. Out of 1,019 participants, gallstone was found in 61 (6%) subjects. Among them, 14 were males and 47 were females (p = 0.16). Within male participants, prevalence was higher below the age of 40 years. In females, prevalence of gallstone disease was found higher below the age 40 years and more marked below 30 years. Total 27 persons had single stone and rest 34 had multiple stones (Table 1).

Gallstone was found more among housewives (42; 68.85%) followed by service holders (9; 14.75%). In this study, middle class socioeconomic group was found to be more affected (30; 49.1%) followed by poorer people (26; 42%) (p = 0.3886). Among the persons having gallstone disease, only three (4.9%) were smokers and 13 (21.31%) used to take betel nut and tobacco leaves.

Among all 61 persons having GSD, seven were underweight, 32 were normal weight, 17 were overweight and five were obese and extremely obese (p = 0.894). The prevalence of GSD in relation to age group and body mass index (BMI) has been shown in Tables 2 and 3 respectively.

## DISCUSSION

Prevalence of asymptomatic GSD in North-East part of Bangladesh in this study was found to be 6% among adults. It is similar to another report from southern coastal region of this country which was 5.4%.^14^ It is higher than the prevalence in Japan^9^ and Thailand,^10^but similar to that of Italy^7^ and Germany.^15^ But, this is lower than that of Taiwan,^8^ Sweden^5^ and Mexico.^6^

**Table 1:** **Table 1:** Relation of gallstone disease with demographic variables

		*Total*		*GB stone*		*Percentage*	
Male		316		14		4.43	
Female		703		47		6.68	
		1019		61		5.98	
*Education*							
Primary and below		528		35		6.62	
Up to HSC		374		20		5.34	
Above		117		6		5.12	
*Occupation*							
Housewife		602		62		10.29	
Student		37		3		8.108	
Service		216		9		4.166	
Business and farming		79		3		3.797	
Day labor and others		60		4		6.66	
*Glucose tolerance*							
Diabetic		63		3		4.76	
Nondiabetic		956		58		6.066	
*Economic class*							
Poor		505		26		5.148	
Middle class		435		30		6.896	
Rich		79		5		6.32	
*Personal habit*							
Smoker		103		3		2.91	
Nonsmoker		916		58		6.33	
Betel nut chewer		166		13		7.83	
Nonbetel nut chewer		853		48		5.627	

**Table 2:** **Table 2:** Prevalence of gallstone disease in different age groups

*Age groups*		*Population*		*GB stone*		*Male*		*Female*	
≤ 30 years		392		23		4		19	
31-40 years		313		17		4		13	
41-50 years		188		10		1		9	
> 50 years		126		11		5		6	

**Table 3:** **Table 3:** Relation of gallstone disease with BMI

*BMI*		*Total* *population*		*Gallstone*		*Percentage*	
Underweight		93		7		7.52	
Normal weight		585		32		5.47	
Overweight		278		17		6.11	
Obese		61		5		8.19	
Extreme obese		1		0		0	

These differences maybe due to differences in ethnicity, geographical environment and food habit and lifestyle. Prevalence of gallstone disease is found higher among women than men in this study. It is consistent with other reports.^1,5-7,16,17^

In this study, prevalence was higher among the people of below 40 years which is not consistent with other reports^11,18,19^ where increasing age was found one of the common risk factor for GSD. The number of participants of higher age group was less in the present communication. Obesity has not been found to be a significant risk factor which is also inconsistent with other reports from our country^14^ and Europe.^11,19^ However, subjects the number of overweight and obese participants were also small. But, other non-European reports^9,11,19^ did not support the association of obesity in the formation of gallstone. This may be due to nature of stone, lifestyle of population and environmental factors. Black stones are related with hemolytic diseases and brown stones are related with stasis and infection in biliary system. Obesity plays role at least in part in formation of cholesterol stones which is infrequent in Asians and common in Europeans.^15^

This study revealed that middle class and poorer people are more affected by gallstones which are consistent with reports from Britain.^20^ But, this differs from the other study in Bangladesh.^14^ It maybe explained as the number of participants of higher socioeconomic group was small in this study.

Prevalence of GSD is found to be significantly higher among housewives, which is consistent with reports from southern part of our country,^14^ and Italy.^20^ But, Swedish reports do not support it.^21^ Smoking habit, habit of betel nut and tobacco leaves chewing, diabetes mellitus and hypertension showed no significant influence on the prevalence of gallstone disease in this study.
